# Effect of Alloying and Reinforcing Nanocomposites on the Mechanical, Tribological, and Wettability Properties of Pulse-Electrodeposited Ni Coatings

**DOI:** 10.3390/mi16020175

**Published:** 2025-01-31

**Authors:** Aashish John, Adil Saeed, Zulfiqar Ahmad Khan

**Affiliations:** NanoCorr, Energy & Modelling (NCEM) Research Group, Bournemouth University, Talbot Campus, Poole BH12 5BB, UK; ajohn@bournemouth.ac.uk (A.J.); asaeed4@bournemouth.ac.uk (A.S.)

**Keywords:** tribology, pulse electrodeposition coating, nanocomposites, wettability, nanotribology, nanohardness, contact angle

## Abstract

Research into the introduction of alloying and reinforcing nanocomposites into nickel (Ni) coatings has been motivated by the need for tribologically superior coatings that will improve energy efficiency. Using pulse electrodeposition, this work investigates the effects of adding cobalt (Co) as the alloying nanoparticle and silicon carbide (SiC), zirconium oxide (ZrO_2_), and aluminium oxide (Al_2_O_3_) as reinforcing nanocomposites to Ni coatings. The surface properties, mechanical strength, nanotribological behaviour, and wettability of these coatings were analysed. Surface characteristics were evaluated by the use of a Scanning Electron Microscope, revealing a grain dimension reduction of approximately ~7–43% compared to pristine Ni coatings. When alloying and reinforcing nanocomposites were added to Ni coatings, nanoindentation research showed that there was an increase in nanohardness of ~12% to ~69%. This resulted in an improvement in the tribological performance from approximately 2% to 65%.The hydrophilic nature of Ni coatings was observed with wettability analysis. This study demonstrates that nanocomposite reinforcement can be used to customise Ni coatings for applications that require exceptional tribological performance. The results point to the use of Ni-Co coatings for electronics and aerospace sectors, with more improvements possible with the addition of reinforcing nanoparticles.

## 1. Introduction

The electrodeposition of metals and alloys on various metallic substrates is widely applied in different domains, including aerospace, mechanical, electrical, automotive, and nuclear industries [1,2,3]. A new era of composites has begun, with finely dispersed nanoparticles being electrochemically deposited within a metallic matrix. Bespoke coating characteristics are achieved by customising electroplating physical properties, types of nano constituents and electrolytic characteristics and are key benefits of electroless pulse coating techniques. Other advantages include low working temperatures, ease of maintenance, and cost-effectiveness [4,5,6,7,8,9,10].

On account of their strong resistance to thermal oxidation and corrosion, Ni coatings have gained much attention. The use of W, Mo, Mn, Cr, and P nano constituents as additives to Ni-based coatings leads to significant tribo performance. Further improvements can be achieved with the addition of reinforcing nanocomposites to the Ni metal matrix [6,11,12,13,14,15,16,17,18,19,20,21,22,23,24]. Ni-Co alloy nanocomposite coatings’ magnetic, catalytic, electrochemical energy storage capacity, high-temperature resistant oxidation (including corrosion), and anti-wear properties are desirable across various industries, including sensors, automotive components, actuators, inductors, aerospace technologies, and supercapacitors [25,26,27,28,29].

The incorporation of nanoparticles into a metal matrix (MMC) through pulse electrodeposition improves the tribological properties of these nanocomposite coatings. MMC variations during the process lead to the optimisation of nanocoatings’ morphological, structural, mechanical, and tribo properties. The influence of Al_2_O_3_ in Ni coatings has been studied by Chen et al. in terms of variation in frequency, which affects the volumetric change in Al_2_O_3_ deposition [30]. An inverse relation between frequency and hardness has been observed. A resultant microstructure variation leads to abrasive wear mode as the key tribo failure mechanism. The addition of Al_2_O_3_ to a coating restricts grain movement, which results in an improvement in overall hardness [4,31,32]. Alumina’s addition has led to enhanced wear resistance due to hardness improvement. The addition of Al_2_O_3_ results in an increase in ductility and, in turn, deformation hardening and resistance, ensuring increased load bearing and wear resistance capacities (Gul et al.) [31]. The addition of alumina was seen to restrict grain growth, which reduces the grain size, leading to an increase in hardness. This mechanism enhanced the tribological properties of nanocomposite coatings [33]. Similar observations were made regarding the hardness of nanocoatings with SiC and ZrO_2_. The addition of these secondary nanocomposites improved the hardness [6,9,34,35,36,37]. Gyftou et al. [35] noticed a decrease in grain size when SiC particles were added. SiC particles demonstrated a higher resistance to wear in coatings due to a similar inverse relation between hardness and frequency, observing an increase to decrease and vice versa. Zhou et al. [9] observed a pore-free, uniform thickness, and defect-free Ni coating with SiC as a reinforcing nanocomposite. Mostafa et al. [36] reported a nodular microstructure for NiP-ZrO_2_ coatings, which helps improve the hardness of coatings.

There have been several research on the variation of physical parameters of Ni pulse electrodeposition coating. Investigation of synergistic effects of alloying element (Co), with various reinforcing MMCs (Al_2_O_3_, SiC, and ZrO_2_). Ni-Co exhibits a unique magnetic property, is a novel approach, which makes it a potential candidate in the electronics industry. The reinforcing MMCs are selected due to their extensive application in mechanical, aerospace and automobile industries. This comprehensive analysis uniquely evaluates structural, mechanical, tribological, and wettability properties, targeting multifunctional applications across the electronics, aerospace, and automotive industries.

## 2. Materials and Methods

Pulse electrodeposition was selected to prepare the composite coating. EN1A was selected as the cathode and nickel plate as the anode, with the cathode being the target material wherein the coating is formed. EN1A steel was selected as the substrate due to its low cost, handiness, and adhesive properties. EN1A was produced as a circular-shaped cathode with a diameter × thickness of 30 mm × 3.5 mm. The following Table 1 provides chemical composition information. The nickel anode was produced in a rectangular shape of 2 mm thickness. Surface conditioning was conducted by using emery paper and water lubrication to obtain a surface areal roughness below 0.05 µm. Furthermore, samples were conditioned ultrasonically with distilled water and acetone to eliminate oxide layers and contaminants present on the surface.

**Figure 1:**
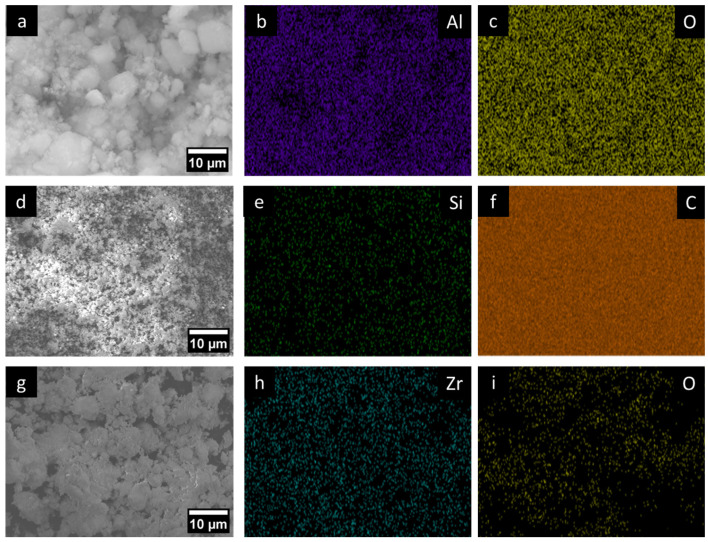
SEM images of MMCs used. (**a**) Al_2_O_3_ (**d**) SiC, and (**g**) ZrO_2_ with their corresponding elemental map, {(**b**,**c**) Al_2_O_3_ (**e**,**f**) SiC and (**h**,**i**) ZrO_2_}.

A modified Watt solution was employed as the electrolyte for the present pulsed electrodeposition coating. For the Ni-Co coating, cobalt sulphate was added to the modified Watt solution as the cobalt source. Aluminium oxide, silicon carbide, and zirconium dioxide were added to the Ni-Co solution as reinforcing MMCs (Figure 1). Nickel sulphate, nickel chloride, boric acid, and cobalt sulphate were purchased from Thermo Scientific (Waltham, MA, USA), Acros Organics (Geel, Belgium), Fisher Scientific (Pittsburgh, PA, USA), and Alfa Aesar (Lancashire, UK), respectively. Aluminium oxide and zirconium dioxide were obtained from Aldrich Chemistry (Dorset, UK) and silicon carbide was obtained from Io-Li-Tec (Heilbronn, Germany). Chemicals were in crystal form with 98% purity. A different electrolyte was prepared for each reinforcing material. The electrolytes were stirred with a magnetic stirrer for 24 hrs and further ultrasonication of 10 kHz was provided for 20 mins before initiating the coating process. The composition of electrolytes is given in Table 2.

A pulse power generator was employed for the current coating. The duty cycle, current density, and frequency were kept constant with those values from earlier studies [38,39,40]. A constant duty cycle of 20% (T_ON_ = 20 ms and T_OFF_ = 80 ms) and current density and frequency at 3 A/dm^2^ and 10 Hz, respectively, were maintained. Constant stirring of the electrolytes was employed during the coating process. A 300 rpm speed was set to facilitate the stirring process, which ensured the turbulent flow of chemicals throughout the coating process. The temperature of the electrolyte was 60 ± 5 °C and the electrolyte pH was 4.2 ± 0.2. The time of coating was set to 60 min. The test configuration is shown in Table 3. Post-electrodeposition, samples were rinsed in an ultrasonic bath containing distilled water and acetone to eliminate residual contaminants. Samples were cleansed in a controlled lab environment.

### 2.1. Characterisation of Nanocrystalline Coating

A Scanning Electron Microscope (SEM) (JEOL, Garden City, UK) was deployed for surface analysis. Nanocrystalline coatings’ constituents were measured by Energy Dispersive X-ray (EDS) (Oxford Instruments, Abingdon, UK) coupled with the SEM.

Samples’ roughness was analysed by utilising White Light Interferometry; the parameters of *Ra*, *Rq*, *Rt* and *Rz* were obtained along with bearing area curves. Ra can be defined as “the arithmetic mean deviation of the sampling length, whereas Sa is defined as the arithmetic mean deviation of the sampling area”. *Rq* is defined as “the root mean square deviation of the assessed sampling length” and *Sq* is “the root mean square deviation of the assessed sampling area”. Rt and St are “the total height of the profile (height between deepest valley and highest peak) of the assessed sampling length and sampling area”, respectively. *Rz* is “the maximum height of the profile in the sampling length”.

$$Ra=\frac{1}{L}\int |z(x)|dx$$

$$Sa=\frac{1}{A}\iint \left|z\left(x,y\right)\right|dx.dy$$

where ‘*L*’ is sampling length, ‘*A*’ is sampling area, and z is the ordinate of the profile curve at a given ordinate or height (x, y) [41,42,43].

### 2.2. Mechanical Characterisation

A 3-faced Berkovich, pyramidal diamond indenter was employed to acquire nanohardness data of coatings (Micromaterials, Wrexham, UK). A total of 15 indents were made on the coating and the values were analysed to determine nanohardness and reduced modulus.

Tribological experiments were conducted using a modified ball-on-plate reciprocating tribometer. Here, 6 mm diameter AISI 52100 chrome steel balls served as the counter face against the coated flat samples. The coated samples were secured within a stationary specimen chamber, while the counter face underwent reciprocating motion at a frequency of 5 Hz. Key experimental parameters were maintained at constant values throughout: a contact load of 2 N, 2000 cycles, a sliding distance of 2 m, and a frequency of 5 Hz. The experiments were conducted in ambient room temperature conditions. Post tribological experiments, wear volume was obtained with the help of a profilometer. The roughness of the wear scar was obtained and analysed. The scars were analysed under SEM to understand the wear mechanisms involved.

To investigate tribological behaviour at the nanoscale, nanotribological wear experiments were performed on the coatings. A spherical indenter with a 28 µm diameter was employed for multi-wear analysis. Three distinct loads were applied: 100 mN, 200 mN, and 300 mN. A total of 12 passes were executed along a 1000 µm track at a scan velocity of 10 µm/s. The initial and final passes served as topography scans, conducted at a minimal load of 0.5 mN. Load application commenced at a distance of 200 µm from the scan initiation point at a loading rate of 4 mN/s. To ensure repeatability, each load condition was replicated three times. Wear depth and scar roughness were subsequently quantified using a profilometer to analyse the nanotribology scars. Scanning electron microscopy (SEM) was utilised for further examination of wear scars’ morphology.

To study the wettability properties of the coatings, they were subjected to wettability analysis. A custom-made setup was used for the wettability analysis, with a droplet size of 7 µL. A DSLR camera was used to capture images of the droplet size, and images were analysed with ImageJ (1.54 g), an open licenced software.

## 3. Results

### 3.1. Surface Morphology

**Figure 2:**
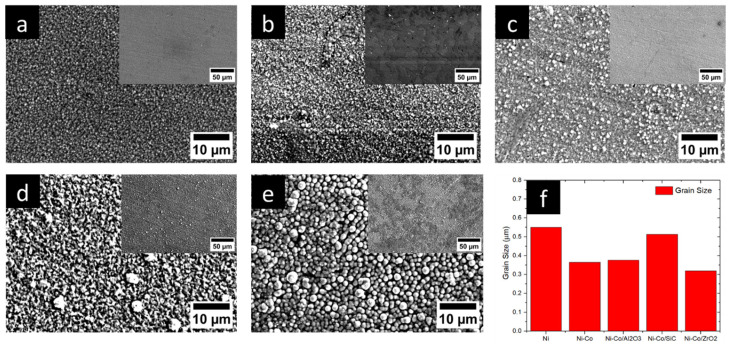
SEM micrographs of the coatings (with low magnification in the inset image): (**a**) Ni, (**b**) Ni-Co, (**c**) Ni-Co/Al_2_O_3_, (**d**) Ni-Co/SiC, (**e**) Ni-Co/ZrO_2_, (**f**) grain size.

The Ni pulse electrodeposition coating with Co as the alloying particle with Al_2_O_3_, SiC, and ZrO_2_ as reinforcing nanocomposites is illustrated in Figure 2. A fine grain structure was observed for Ni-coated surfaces. A globular grain structure was detected for the Ni coating with a 0.55 µm average grain size. The addition of Co particles as the alloying element caused the grain size to decrease to 0.36 µm. A further decrease in grain size could be observed when reinforcing nanoparticles were introduced. With the addition of Al_2_O_3_, SiC, and ZrO_2_ as the reinforcing agents, the grain size further decreased to 0.37 µm, 0.51 µm, and 0.31 µm, respectively. Incorporating alloying and reinforcing MMCs into Ni coatings resulted in a notable reduction in grain size, ranging from ~7% to ~43%. There are several explanations for why adding alloying element and MMCs causes the grain size to decrease. Firstly, the presence of Co as an alloying element resulted in increased nucleation sites. An increase in nucleation regions yielded decreased grain size and grain growth [44,45]. Increasing the pulse-off duration facilitated greater replenishment of alloying elements and metallic matrix composites (MMCs) in the vicinity of the substrate. Subsequently, during the pulse-on phase, these replenished nanoparticles were more readily adsorbed onto the substrate. This enhanced adsorption resulted in an increased density of nucleation sites and consequently a reduction in the overall grain size. It was espied earlier that with a decrease in duty cycle, a finer but more compact grain structure can be obtained [46,47,48]. The influence of grain size on nanohardness and nanotribological properties is explored.

The mechanism of the particle co-deposition of Co and MMCs can be explained with Gugliemi’s model. The Guglielmi model describes the transfer mechanism of nanoparticles from an electrolytic solution to a substrate, forming a composite coating. According to this model, the process involves two key stages: loose adsorption, where the particles are loosely adsorbed to the cathode surface by Van der Walls forces, and strong adsorption, where under the electric field and electrochemical reactions, the particles transition to the strong adsorption state [49,50].

**Figure 3:**
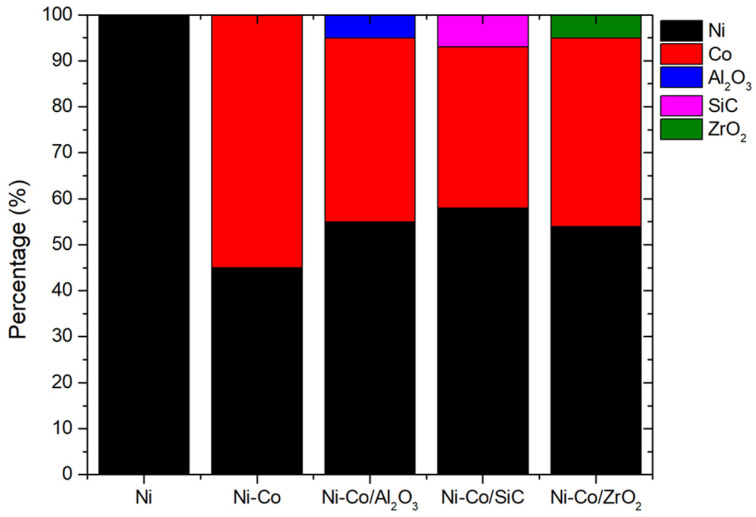
EDS analysis of pulsed electrodeposition coatings of Ni, Ni-Co alloy, and Ni-Co alloy with reinforced materials (Ni-Co/Al_2_O_3_, Ni-Co/SiC, and Ni-Co/ZrO_2_).

To understand the elemental configuration associated with the nanocomposite coating, EDS analyses were obtained. Figure 3 depicts the coatings’ EDS mapping. For the nickel coating, the coating consisted of only Ni, which was 100%. The amount of Ni decreased when Co was added as an alloying element, to 57%, and the remainder was Co, 43%. A further decrease could be observed with the addition of reinforcing agents. When Al_2_O_3_ was added to the coating as an MMC, the Ni and Co presence decreased to 55% and 40%, respectively, with 5% Al_2_O_3_. Ni-Co/SiC and Ni-Co/ZrO_2_ had similar decreases in Ni and Co content. Ni-Co/SiC had 58% of Ni and 35% of Co, with SiC having 7%. Ni-Co/ZrO_2_ also exhibited a similar decline in Ni and Co, 54% and 41%, respectively, with ZrO_2_ 5%.

### 3.2. Surface Roughness

**Figure 4:**
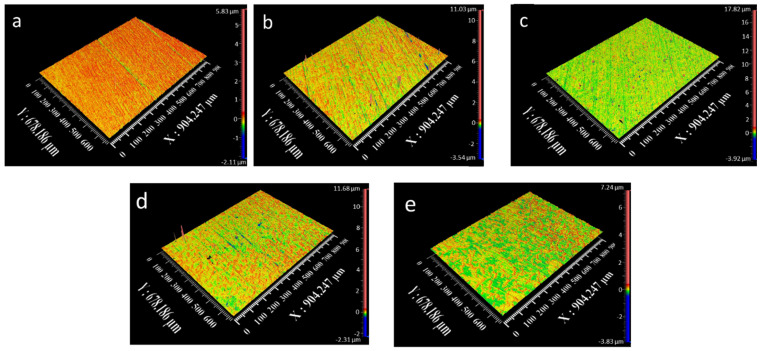
Surface profiles of (**a**) Ni, (**b**) Ni-Co, (**c**) Ni-Co/Al_2_O_3_, (**d**) Ni-Co/SiC, and (**e**) Ni-Co/ZrO_2_.

**Figure 5:**
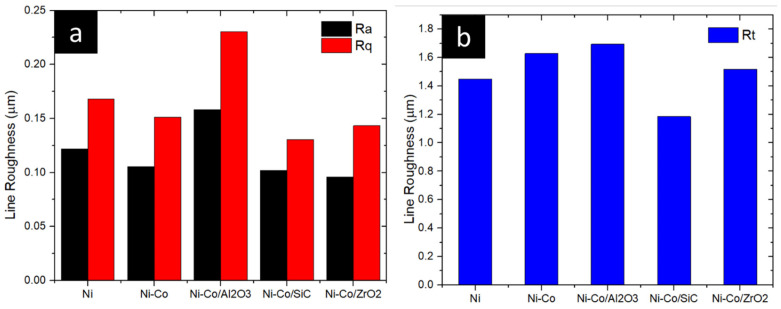
Line roughness of pulse electrodeposition coating. (**a**) Ra and Rq (**b**) Rt.

Profilometer images of the coatings are shown in Figure 4. Line roughness parameters, including *Ra*, *Rq*, *Rz*, and *Rt*, were evaluated for the nanocrystalline coatings and are presented in Figure 5. The Ni-coating exhibited a line roughness of 0.122 µm. Roughness values for Ni-Co, Ni-Co/Al_2_O_3_, Ni-Co/SiC, and Ni-Co/ZrO_2_ were determined to be 0.105 µm, 0.158 µm, 0.096 µm, and 0.102 µm, respectively.

To gain a more precise understanding of line roughness, the root-mean-square (RMS) line roughness was calculated, revealing a trend consistent with the overall line roughness. Ni-Co/Al_2_O_3_ exhibited the highest RMS roughness at 0.230 µm, followed by Ni, Ni-Co, Ni-Co/SiC, and Ni-Co/ZrO_2_ with RMS values of 0.168 µm, 0.151 µm, 0.143 µm, and 0.130 µm, respectively.

Maximum peak-to-valley height (Rt) was measured as 1.447 µm, 1.628 µm, 1.694 µm, 1.517 µm, and 1.184 µm for Ni, Ni-Co, Ni-Co/Al_2_O_3_, Ni-Co/SiC, and Ni-Co/ZrO_2_, respectively. Similarly, Rz values were obtained, displaying a trend analogous to the RMS line roughness. *Rz* values were recorded as 1.258 µm, 1.079 µm, 1.368 µm, 0.959 µm, and 0.859 µm, respectively.

**Figure 6:**
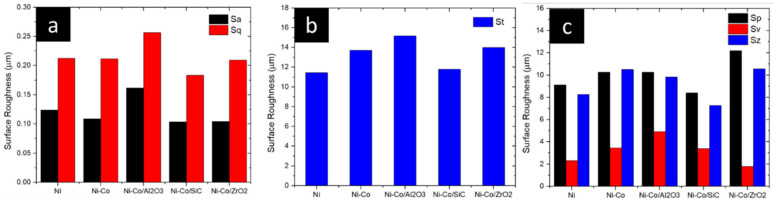
Surface roughness of pulse electrodeposition coatings. (**a**) Sa and Sq, (**b**) St and (**c**) Sp, Sv, Sz.

To gain a comprehensive understanding of surface roughness, areal roughness analysis was conducted, providing a two-dimensional assessment of surface topography, as depicted in Figure 6. The areal roughness of the Ni coating was measured at 0.124 µm, which subsequently varied to 0.108 µm, 0.162 µm, 0.104 µm, and 0.103 µm for Ni-Co, Ni-Co/Al_2_O_3_, Ni-Co/SiC, and Ni-Co/ZrO_2_, respectively.

Consistent with previous observations, the root-mean-square (Sq) roughness was also evaluated to provide a more in-depth analysis of surface texture. Sq values were recorded as 0.212 µm, 0.211 µm, 0.256 µm, 0.209 µm, and 0.183 µm, respectively. The incorporation of alloying elements and reinforcing nanocomposites into the Ni coating significantly altered the surface morphology.

Maximum peak-to-valley height of the entire surface, denoted as St, was also considered. St values were observed to be 11.456 µm, 13.720 µm, 15.175 µm, 14.006 µm, and 11.797 µm, respectively. In line with the observed surface roughness trends, an increase in St was noted for the coatings incorporating reinforcing and alloying agents.

To determine average distance between peaks and valleys, average peak-to-valley height (Sz) was analysed. Sz values were found to be 8.27 µm, 10.49 µm, 9.83 µm, 10.55 µm, and 7.27 µm, respectively.

**Figure 7:**
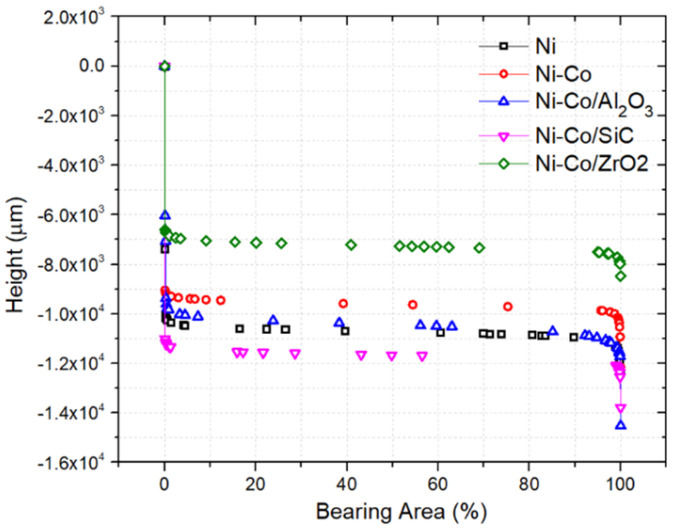
Bearing area curves of pulse electrodeposition coatings.

The bearing area curves of the nanocomposite coatings were analysed and are shown in Figure 7. It is seen that the *Rk* value was highest for Ni and was almost similar to Ni-Co/Al_2_O_3_. Following that, all the nanocoating MMCs had a lower *Rk* value. The *Rk* value signifies the core roughness depth over the sampling area. The *Rk* value was efficacious over the tribological properties of the coating, which is discussed in later sections. The MR1 and MR2 values were analysed from the bearing area curves of the nanocomposite coatings. It was observed that MR1 values were similar for all the nanocomposite coatings. However, while analysing MR2, Ni and Ni-Co/Al_2_O_3_ had lower values and all other coatings had a higher MR2 value. The influence of bearing area curve and roughness on nanotribological properties is explored further with a nanotribometer.

### 3.3. Nanohardness

**Figure 8:**
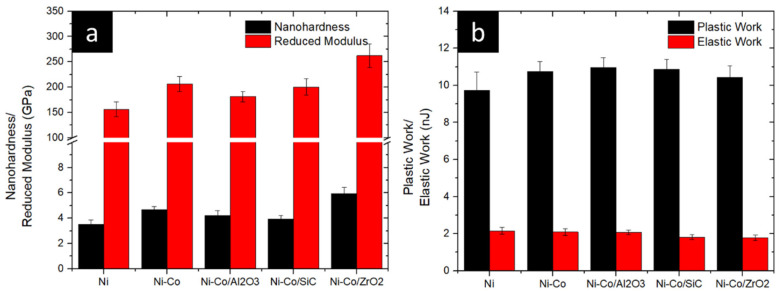
Effect of alloying and reinforcing composites of Ni coating on (**a**) nanohardness and reduced modulus and (**b**) plastic–elastic work.

Nanoindentation tests were conducted to investigate nanocoatings’ mechanical properties. Figure 8 illustrates the influence of alloying and reinforcing nanoparticles on nanohardness. The Ni coating exhibited a hardness of 3.51 GPa. Subsequently, an increase in nanohardness was observed in Ni-Co, Ni-Co/Al_2_O_3_, Ni-Co/SiC, and Ni-Co/ZrO_2_, attributed to the presence of reinforcing nanomaterials.

The highest hardness, 5.95 GPa, was achieved for the Ni-Co/ZrO_2_ coating, where ZrO_2_ served as the reinforcing agent. This represents a substantial 66.51% enhancement compared to the base Ni coating. Conversely, the lowest hardness, 3.93 GPa, was observed for the SiC-reinforced coating.

The nanohardness of Ni-Co and Ni-Co/Al_2_O_3_ was determined to be 4.69 GPa and 4.22 GPa, respectively. The incorporation of Co into the Ni matrix resulted in a 33.61% increase in nanohardness compared to the pristine Ni coating.

In conclusion, the addition of alloying elements and reinforcing nanocomposites to the Ni coating led to a notable enhancement in hardness, ranging from approximately 11% to 69%.

In addition to the above, the reduced modulus of the Ni-based nanocomposite coating was also obtained. The reduced modulus of the pure Ni coating was 156.32 GPa, which was the lowest among all the coatings. The reduced modulus of Ni-Co with alloying reagent was 206.13 GPa and with the additions of reinforcing composites Ni-Co/Al_2_O_3_, Ni-Co/SiC and Ni-Co/ZrO_2_ having reduced moduli of 181.51 GPa, 200.25 GPa, and 262.24 GPa, respectively. With the addition of reinforcing and alloying coating chemicals, the reduced modulus changed.

The hardness of metal matrix composites is influenced primarily by grain refining, dispersion strengthening, and the hardness of the metal matrix. With the addition of Co, nucleation sites increased, which caused an increase in grains, resulting in a decrease in grain size. A refined grain size will hinder the movement of grains, which will increase hardness. A further change in nanohardness was observed with the addition of reinforcing nanocomposites. The metal matrix deposited can influence the hardness in two ways. Firstly, by dispersion strengthening, the alloying elements and MMCs present in the coating will hinder the movement of the grains. This hindered movement will cause an increase in hardness. Secondly, the hardness of incorporated MMCs will influence the hardness of the coating. In the present study, Al_2_O_3_, SiC, and ZrO_2_ were the reinforcing MMCs. The hardness of reinforcing MMC particles will increase the hardness of the overall coating surface. Therefore, the synergistic effect of reduced grain size and hardness of the reinforcing particles will increase the hardness of the coatings. The influence of nanohardness on the tribological properties of coatings is explored with a nanotribometer and ball-on-plate tribometer.

### 3.4. Sliding Wear Behaviour

**Figure 9:**
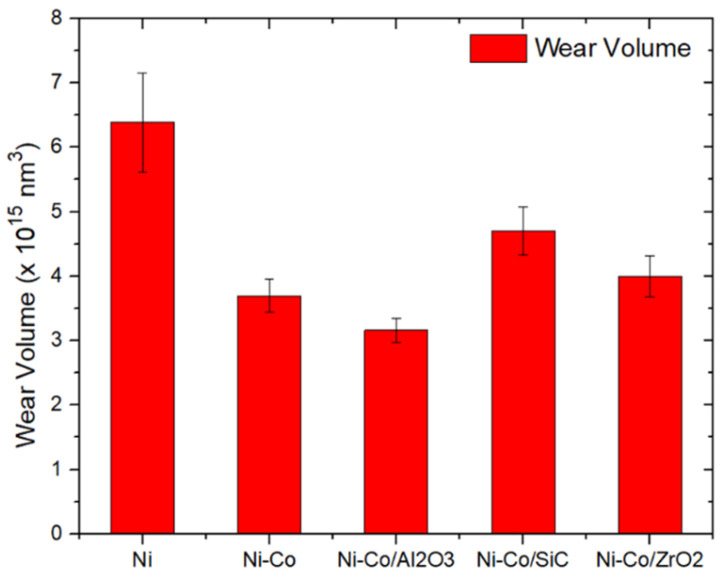
Effect of alloying and reinforcing composites of Ni coating over tribological properties.

The wear volume of coatings is shown in Figure 9. The pure Ni coating had a wear volume of 6.38 × 10^15^ nm^3^, which was followed by Ni-Co/SiC having a wear rate of 4.70 × 10^15^ nm^3^. Ni-Co/ZrO_2_ and Ni-Co had wear rates of 4.00 × 10^15^ nm^3^ and 3.69 × 10^15^ nm^3^, respectively, followed by Ni-Co/Al_2_O_3_, which had the best wear rate of 3.16 × 10^15^ nm^3^. Therefore, it can be concluded that Ni-Co/Al_2_O_3_ had the lowest wear rate of 3.16 × 10^15^ nm^3^, which had Al_2_O_3_ as the reinforcing agent. The coating with the least wear resistance was the Ni coating. The addition of Co to Ni was seen to improve the wear properties. The reinforcing agents Al_2_O_3_, SiC, and ZrO_2_ helped in increasing the wear resistance of the coating.

Ni-Co/Al_2_O_3_ having the highest wear resistance is due to Al_2_O_3_ which acts as a lubricant. After the initial cycles, Al_2_O_3_ detached from the coating surface, which acted as a lubricant. Three-body interaction was initiated, with Al_2_O_3_ acting as a lubricant. SiC also exhibited similar properties, with a decreased wear rate. However, the lower hardness of Ni-Co/SiC resulted in the removal of the coating layer at a higher rate, resulting in a higher wear rate when compared to Ni-Co/Al_2_O_3_. Ni-Co/ZrO_2_, which had ZrO_2_ as the reinforcing agent, was also seen to have higher wear resistance. ZrO_2_ is known to have higher hardness by itself, which helped in improving the wear rate.

**Figure 10:**
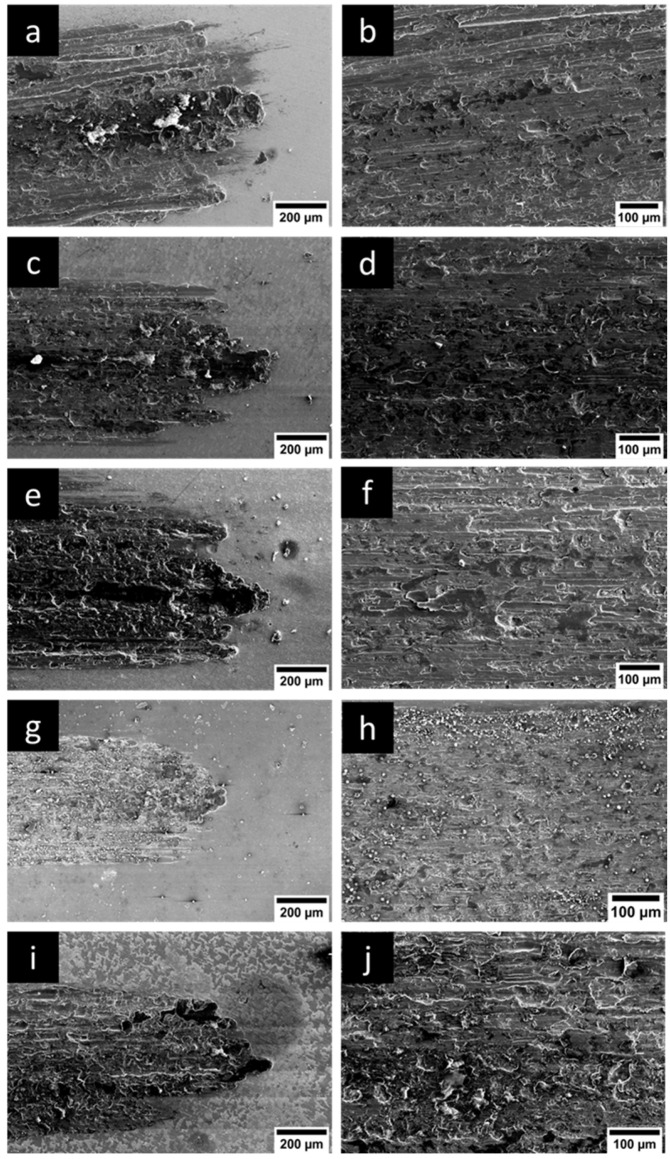
Wear morphologies of Ni nanocomposites with different alloying and reinforcing composites. (**a**,**c**,**e**,**g**,**i**) Wear morphology at edges and (**b**,**d**,**f**,**h**,**j**) wear morphology and centre of wear scar. (**a**,**b**) Ni, (**c**,**d**) Ni-Co, (**e**,**f**) Ni-Co/Al_2_O_3_, (**g**,**h**) Ni-Co/SiC, (**i**,**j**) Ni-Co/ZrO_2_.

SEM images of wear scars were obtained and are provided in Figure 10 to understand prevailing wear mechanisms. Abrasive wear and oxidation wear were seen, along with delamination and adhesive wear in the case of coatings. The pitting of nanoparticles was also observed on wear tracks. With the Ni-Co coating, abrasive wear along with microploughing and delamination wear was observed. For the Ni-Co/Al_2_O_3_ coating, delamination wear was dominant along with microploughing and abrasion. However, in Ni-Co/SiC, oxidation wear was the predominant mechanism, with micropitting and delamination also present. Similar observations were observed in Ni-Co/ZrO_2_ as well. These findings are consistent with previous research, demonstrating alignment with earlier studies. Therefore, it can be concluded that alloying and reinforcing elements improved the tribological attributes of Ni composite coatings.

### 3.5. Nanotribology Analysis

**Figure 11:**
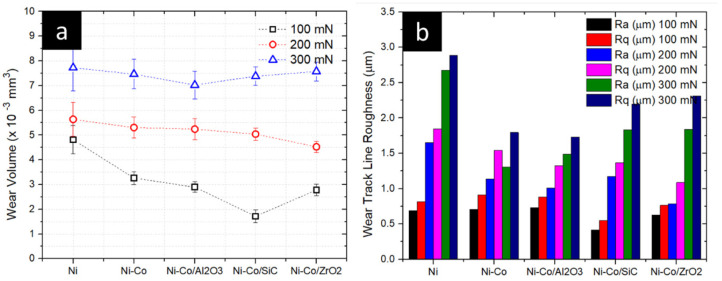
Effect of alloying and reinforcing composites of Ni coating over (**a**) nanotribological properties, (**b**) wear line roughness.

**Figure 12:**
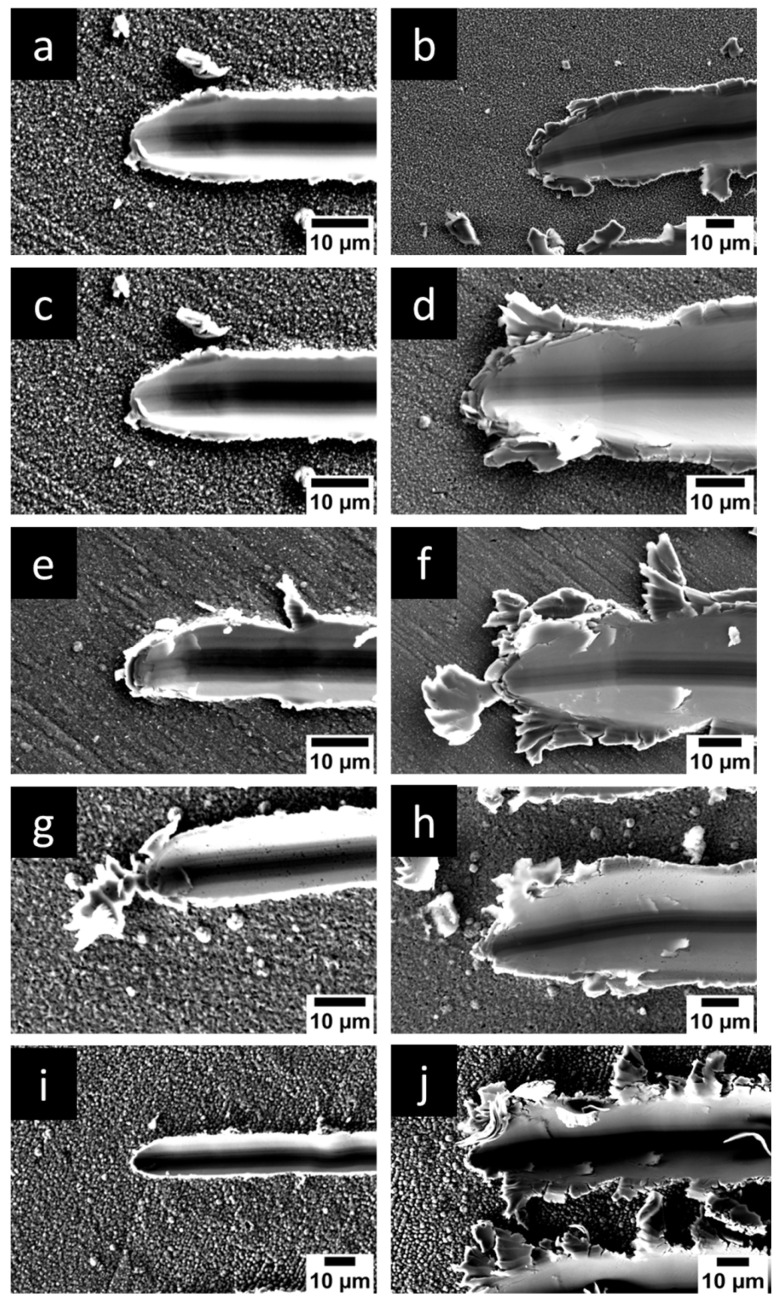
Nanowear morphologies of Ni nanocomposites with different alloying and reinforcing composites. (**a**,**c**,**e**,**g**,**i**) Wear morphology at edges and (**b**,**d**,**f**,**h**,**j**) wear morphology and centre of wear scar. (**a**,**b**) Ni, (**c**,**d**) Ni-Co, (**e**,**f**) Ni-Co/Al_2_O_3_, (**g**,**h**) Ni-Co/SiC, (**i**,**j**) Ni-Co/ZrO_2_.

The nanotribological analysis was performed to gain a deeper understanding of the tribological properties on a nano-level load. The load was varied from 100 mN to 300 mN and wear volume was analysed. The wear volumes of various nanocomposite coatings are displayed in Figure 11, with SEM images of wear scars in Figure 12. At the load of 100 mN, Ni-Co/SiC exhibited the best wear properties with a wear volume of 1.71 × 10^−3^ mm^3^, which was accompanied by Ni-Co/ZrO_2_, Ni-Co/Al_2_O_3_, Ni-Co, and Ni, with a wear volume of 2.78 × 10^−3^ mm^3^_,_ 2.89 × 10^−3^ mm^3^, 3.26 × 10^−3^ mm^3^, and 4.81 × 10^−3^ mm^3^. When the load was increased to 200 mN, Ni-Co/ZrO_2_ had a wear volume of 4.50 × 10^−3^ mm^3^, which was followed by Ni-Co/SiC, Ni-Co/Al_2_O_3_, Ni-Co, and Ni with a wear volume of 5.04 × 10^−3^ mm^3^, 5.24 × 10^−3^ mm^3^, 5.30 × 10^−3^ mm^3^, and 5.63 × 10^−3^ mm^3^. Furthermore, when load was increased to 300 mN, the wear volume was best for Ni-Co/Al_2_O_3_, which was followed by Ni-Co/SiC, Ni-Co/ZrO_2_, Ni-Co, and Ni with a wear volume of 7.02 × 10^−3^ mm^3^, 7.98 × 10^−3^ mm^3^, 7.46 × 10^−3^ mm^3^, 7.57 × 10^−3^ mm^3^, and 7.72 × 10^−3^ mm^3^, respectively. It can be espied from the analysis that during all the loads, the nanocoating with reinforcing nanocomposites had nanowear superior to that of the Ni and Ni-Co coatings.

By classical Archard’s law,

$$Q\propto \frac{WL}{H}$$

The wear volume (*Q*) is governed by the equation *Q* α *W*∗*L*/*H*, where *W* = total normal load, *L* = sliding distance, and *H* = hardness of contacting surface. This equation reveals an inverse relationship between wear volume and surface hardness. Nanocomposite coatings, such as Ni coatings incorporating alloying and reinforcing agents, exhibit enhanced hardness. This increased hardness translates to a reduced wear rate for the nanocoating. Notably, the addition of reinforcing agents to Ni-Co coatings has been observed to significantly improve their tribological properties.

Surface roughness significantly influences the wear rate of coatings. The incorporation of alloying and reinforcing agents into pure Ni coatings often results in increased surface roughness, characterised by the presence of more pronounced peaks. During initial tribological interactions, these peaks engage with the counter-surface, leading to localised high-contact pressures. This elevated pressure can accelerate material removal. Conversely, smoother surfaces with lower roughness exhibit a larger contact area, distributing the load more evenly and consequently reducing contact pressure. This diminished pressure translates to a lower rate of material removal. Therefore, the synergistic effect of enhanced nanohardness and optimised surface roughness is crucial in achieving superior wear resistance in these coatings.

### 3.6. Wettability

**Figure 13:**
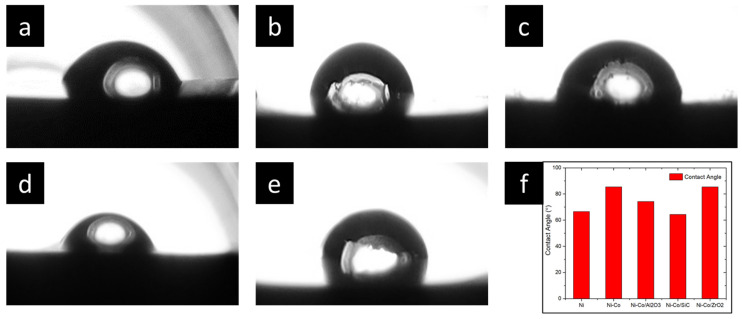
Effect of alloying and reinforcing composites of Ni coating over contact angle properties. (**a**) Ni, (**b**) Ni-Co, (**c**) Ni-Co/Al_2_O_3_, (**d**) Ni-Co/SiC, (**e**) Ni-Co/ZrO_2_, (**f**) comparisons.

Contact angles of nanocomposite coatings were measured with a customised setup camera, and the images were analysed with ImageJ, an open licence software. The correlation between the hydrophilic properties of various nanocomposite coatings was analysed (Figure 13). The hydrophilic properties were exhibited by all the nanocomposite coatings. Ni-Co/Al_2_O_3_ had the best hydrophilic property among all the composite coatings with a contact angle of 64.32°. This was followed by Ni and Ni-Co/Al_2_O_3_ with a contact angle of 66.53° and 74.30°, respectively. However, Ni-Co and Ni-Co/ZrO_2_ had almost similar hydrophilic properties with a contact angle of 85.50° and 85.43°, respectively.

Based on the findings from contact angle measurements, it is evident that the nanocomposite coatings exhibited hydrophilic properties. Traditionally, surface roughness is a key factor influencing contact angle properties, as elucidated by Cassie–Baxter and Wenzel theories. However, contrary to expectations, the roughness in this study did not significantly impact the contact angle properties of the coating. Instead, the dominant influence stemmed from the surface energy of the nanoparticles and nanocomposites. Notably, metals are known to possess higher surface energy, and this characteristic was effectively transferred to the coating material. Consequently, all the pulse electrodeposited coatings in this study demonstrated hydrophilic properties.

## 4. Conclusions

The pulse electrodeposition of Ni coatings, with Co as an alloying element and reinforced by Al_2_O_3_, SiC, and ZrO_2_ nanomaterials, was successfully performed. The process exhibited a synergistic effect between the alloying element and reinforcing nanocomposites, significantly enhancing the coating properties. The following conclusions were made:

- Grain size reduction of ~7%–~43% with the incorporation of alloying and reinforcing nanocomposites.
- Improved surface characteristics, as evidenced by surface roughness and bearing area curve analyses.
- Enhanced coating hardness, with increases of ~11%–~69% when compared with the Ni coating, and with Ni-Co/ZrO_2_ achieving the highest hardness of 5.95 GPa.
- Superior wear resistance, with Ni-Co/Al_2_O_3_ demonstrating a ~51% improvement over pure Ni coatings.
- Tribological property enhancements, with improvements ranging from ~2% to ~65%, confirmed through nanotribological assessments.
- Hydrophilic behaviour in all coatings, with Ni-Co/ZrO_2_ exhibiting the highest contact angle and Ni-Co/SiC, the lowest.

## Figures and Tables

**Table 1:** Chemical composition of EN1A steel substrate.

C	Si	Mn	P	S
0.09	0.25	0.91	0.71	0.5

**Table 2:** Electrolyte composition.

Chemical Constituent	Concentration (g/L)	Property
Nickel Sulphate (NiSO_4_∙6H_2_O)	265	Watts Solution/Nickel Source
Nickel Chloride (NiCl_2_∙6H_2_O)	48	
Boric Acid (H_3_BO_3_)	31	
Cobalt Sulphate (CoSO_4_∙6H_2_O)	40	Cobalt Source
Aluminium Oxide (Al_2_O_3_)	10	Al_2_O_3_ Source
Silicon Carbide (SiC)	10	SiC Source
Zirconium Dioxide (ZrO_2_)	10	ZrO_2_ Source

**Table 3:** Pulse electrodeposition coating conditions.

Condition	Parameter
Current Density (A/dm^2^)	3
Duty Cycle (%)	20
Time (min)	60
Temperature (°C)	60
Stir Speed (rpm)	300
pH	4.2 ± 0.2
Cathode	EN1A
Anode	Nickel Plate

## Data Availability

All relevant data is included in this paper.
